# Concerning Dental Colleges

**Published:** 1881-07

**Authors:** 


### CONCERNING DENTAL COLLEGES.

The subject of dental education has elicited considerable discussion in the *American Dental Association* for several years past. No very definite action has ever been had upon the subject till the last annual meeting, when the following was unanimously adopted:

"RESOLVED, That in order to secure representation in this Association, dental colleges must, subsequent to October 1, 1881, require all students entering therein to take two full courses of lectures previous to coming forward for examination and graduation, and must also state these conditions in their next annual announcement."

This would seem to be definite, and yet some student writes every few days that he is promised graduation upon attending one course of lectures, by some of our reputable dental colleges.

Now do some proposed students falsify or prevaricate, or are there some of our colleges that propose to ignore or disregard the action of the American Dental Association? Can any afford to do that? And especially since the profession would suffer by such a course.

Some of the colleges have made the statement in their announcements, as required in the resolution. I do not know whether all have or not.

The resolution embodies the belief and conviction of every true and intelligent friend of the profession in the country. Of course, any college may refuse to conform to the requirement of the Association, but in the outcome nothing would be gained, but much lost. And no members of a college which should take such a stand could consistently apply for membership, or remain in the Association as active members while concurring in such non-compliance.

The truth is our colleges should be uniform in their requirements for entrance, and for graduation. There should be no such thing as competition for students, except that which would arise from a desire and earnest efforts, to give the most thorough professional preparation. Let the emulation be in this direction, and this only, then the best results will be attained.

